# Effects of Probiotics on Intestinal Mucosa Barrier in Patients With Colorectal Cancer after Operation: Meta-Analysis of Randomized Controlled Trials

**DOI:** 10.1097/MD.0000000000003342

**Published:** 2016-04-18

**Authors:** Dun Liu, Xiao-Ying Jiang, Lan-Shu Zhou, Ji-Hong Song, Xuan Zhang

**Affiliations:** From the Fujian Medical University, Fuzhou, Fujian (DL, X-YJ, J-HS, XZ); and The Second Military Medical University, Shanghai, China (L-SZ).

## Abstract

Many studies have found that probiotics or synbiotics can be used in patients with diarrhea or inflammatory bowel disease for the prevention and treatment of some pathologies by improving gastrointestinal barrier function. However, there are few studies availing the use of probiotics in patients with colorectal cancer. To lay the foundation for the study of nutritional support in colorectal cancer patients, a meta-analysis has been carried out to assess the efficacy of probiotics on the intestinal mucosa barrier in patients with colorectal cancer after operation.

To estimate the efficacy of probiotics on the intestinal mucosa barrier in patients with colorectal cancer after operation, a meta-analysis of randomized controlled trials has been conducted.

Databases including PubMed, Ovid, Embase, the Cochrane Central Register of Controlled Trials, and the China National Knowledge Infrastructure have been searched to identify suitable studies. Stata 12.0 was used for statistical analysis, and sensitivity analysis was also conducted. Six indicators were chosen to evaluate probiotics in protecting the intestinal mucosa barrier in patients with colorectal cancer. Ratios of lactulose to mannitol (L/M) and *Bifidobacterium* to *Escherichia* (B/E), occludin, bacterial translocation, and levels of secretory immunoglobulin A (SIgA), interleukin-6 (IL-6), and C-reactive protein (CRP) were chosen to evaluate probiotics in protecting the intestinal mucosa barrier in patients with colorectal cancer.

Seventeen studies including 1242 patients were selected for meta-analysis, including 5 English studies and 12 Chinese studies. Significant effects were found in ratios of L/M (standardized mean difference = 3.83, *P* = 0.001) and B/E (standardized mean difference = 3.91, *P* = 0.000), occludin (standardized mean difference = 4.74, *P* = 0.000), bacterial translocation (standardized mean difference = 3.12, *P* = 0.002), and levels of SIgA (standardized mean difference = 2.91, *P* = 0.004) and CRP (standardized mean difference = 4.21, *P* = 0.000), but no significant effects were found for levels of IL-6 (standardized mean difference = 1.33, *P* = 0.184).

Probiotics can effectively protect the intestinal mucosa physical and biological barrier in patients with colorectal cancer after operation. However, to evaluate the protective effect on intestinal mucosal barrier, further studies on the type and concentration of the probiotics, duration of therapy, and the therapeutic route are required.

## INTRODUCTION

Colorectal cancer is the third most commonly diagnosed cancer in men and the second in women, with about 1.4 million cases and 693,900 deaths occurring in 2012. The incidence of colorectal cancer is increasing in several Asian and Eastern European countries in recent years. The prevalence of risk factors for colorectal cancer includes unhealthy diet, obesity, and smoking.^1^ It is predicted that the new cases of colorectal cancer in China would increase year by year.^2^ Surgery is widely accepted as the cornerstone for curative treatment.^3^ However, surgical trauma and preoperative preparation strategies, such as mechanical bowel preparation, can upset the intestinal microbial balance, deteriorate the gut barrier function, aggravate systemic inflammation, and restrain the immune function, thus resulting in postoperative infections.^3^ As live microorganisms, probiotics can confer a health benefit on the host when administered in adequate amounts.^4^ Moreover, it is also found in some studies that the use of probiotics/synbiotics for the prevention and treatment of some pathologies is effective via improving gastrointestinal barrier function, modifying luminal secretion, affecting epithelial cell proliferation, regulating gut microbiota and immunity, inhibiting bacterial translocation, reducing exposure to toxins, and decreasing the risk of complications.^4–9^ A meta-analysis indicated that the use of prosynbiotics as prophylaxis in patients undergoing colorectal resection is a promising preventive measure that may decrease infection morbidity, the incidence of diarrhea, and gastrointestinal symptoms, (eg, abdominal cramping and flatulence).^10^ Therefore, a meta-analysis has been performed to assess the efficacy of probiotics on the intestinal mucosa barrier in patients with colorectal cancer after operation by reviewing the literature and analyzing the available data.

## MATERIALS AND METHODS

### Literature Search Strategy

Electronic databases including PubMed, Ovid, Embase, Cochrane Central Register of Controlled Trials and China National Knowledge Infrastructure have been searched since database establishment time till April 2015 for the following terms:

1. Prebiotic OR probiotic OR synbiotic;
2. Colorectal neoplasms OR colorectal cancer;
3. Operation OR surgery.

Studies conducted on human subjects have been searched with the restriction that they were written in English and Chinese. Reference lists of reviews and retrieved articles were searched manually at the same time. Abstracts or unpublished reports were not considered.

### Inclusion and Exclusion Criteria

Clinical randomized controlled trials (RCTs) of colorectal cancer patients with elective colorectal surgery were included. The trials evaluated the use of probiotics or synbiotics at the perioperative period. Furthermore, when duplicated articles were reported by the same institution, either the better quality or the most recent publication was included, unless the endpoints were mutually exclusive or were measured at different time intervals. The exclusion criteria were:

1. Patients mainly treated with chemotherapy or radiotherapy
2. Animal studies
3. Lack of approval of local ethics committees
4. Incomplete outcome data
5. Undetermined study type

### Data Collection and Validity Assessment

Data were extracted independently by 2 of the authors (LD and J-HS), who consulted each other to solve any disagreements; when a consensus could not be reached, a third reviewer (X-YJ) would take part in the discussion as referee.

In addition, the methodological quality of the studies included in the meta-analysis was scored using the Jadad scale, which is a 5-point quality scale defining low-quality studies as having a score of <3 and high-quality studies as having a score of ≥3. However, in view of the number of studies identified, low-quality tests, scoring <3 points, were not removed.

### Statistical Analysis

Statistical analyses were conducted using Stata 12.0 software. A value of *P* <0.05 was considered statistically significant. Dichotomous variables were presented as odds ratio with 95% confidence interval. For continuous variables, the standardized mean difference was used if the units were not identical. Statistical heterogeneity was checked using the *χ*^2^ test, and the extent of inconsistency was assessed by the *I*^2^ statistic.

The meta-analysis was conducted using a fixed-effects model when there was no heterogeneity in the trial results. Otherwise, when heterogeneity was found, sensitivity analysis and the random-effects model were used instead. The reliability of the results was greatly increased if the sensitivity analysis did not essentially change the results of meta-analysis.

## RESULTS

### Study Characteristics

**FIGURE 1:**
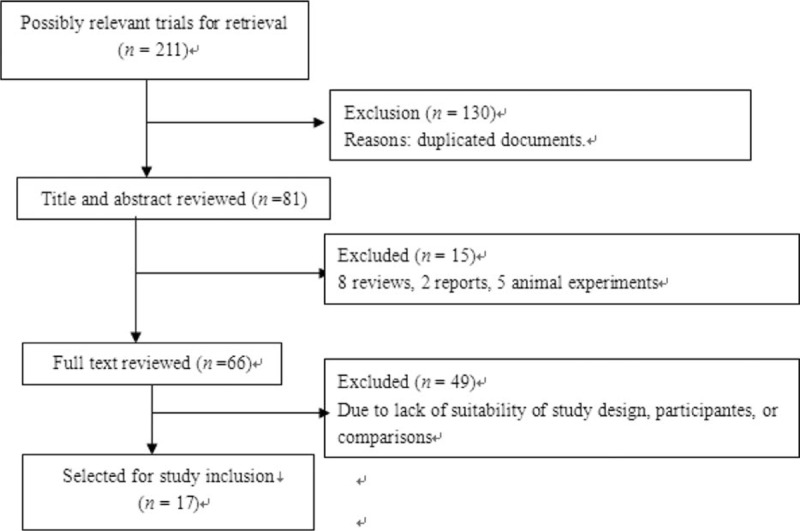
Study flow diagram.

A total of 211 relevant trials have been identified according to predefined search strategy. Only 17 studies (5 in English, 12 in Chinese), involving 1242 patients,^11–27^ were considered to meet the inclusion criteria in this meta-analysis (Figure 1).

In 17 RCTs, the baseline characteristics of the included patients (age, sex, body mass index, type of operation, etc) were compared. There were no statistically significant differences between groups. Characteristics of the studies included in the meta-analysis are presented in Table 1.

**TABLE 1:**
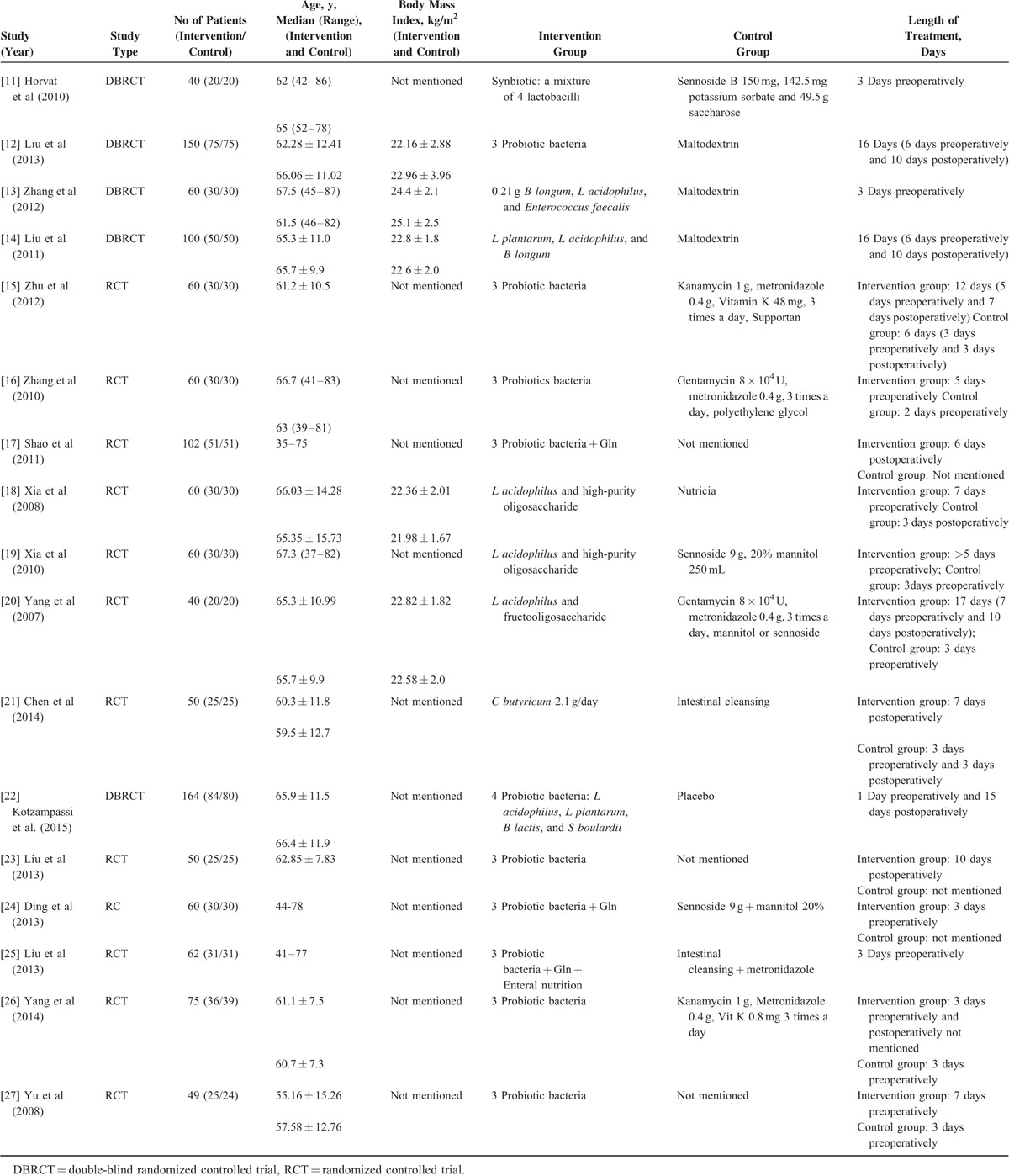
Clinical Characteristics of Included Studies

Seven studies focused only on the preoperative use of probiotics or synbiotics, 8 studies considered preoperative plus postoperative treatment, and 2 studies concentrated on postoperative use only (Table 1).

### Quality Assessment

Five of 17 studies were adequate in random allocation; 4 studies were double-blinded. Furthermore, 5 studies had a Jadad score ≥3 (Table 2 Jadad scale assessment).

**TABLE 2:**
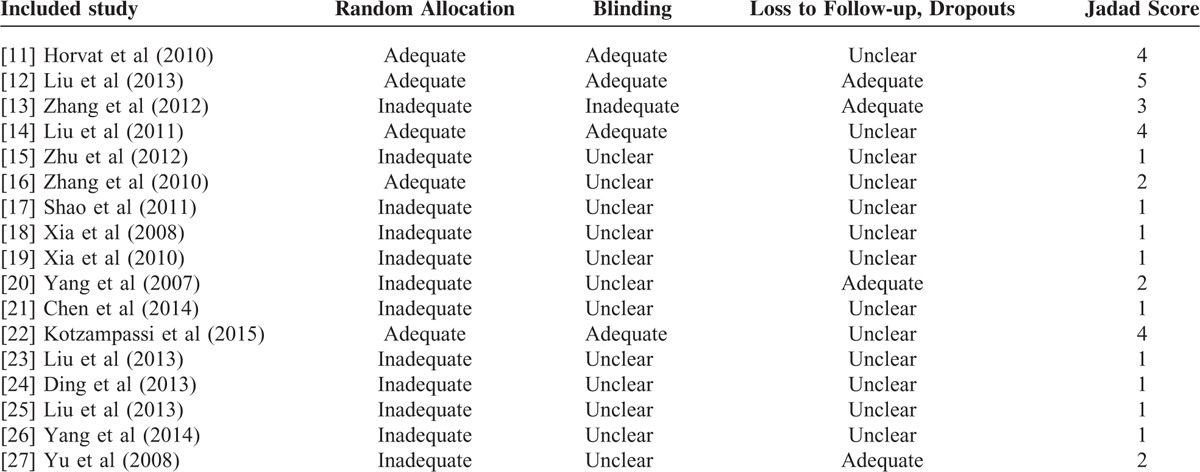
Jadad Scale Assessment

### Synthesis of Results

#### Lactulose/Mannitol

**FIGURE 2:**
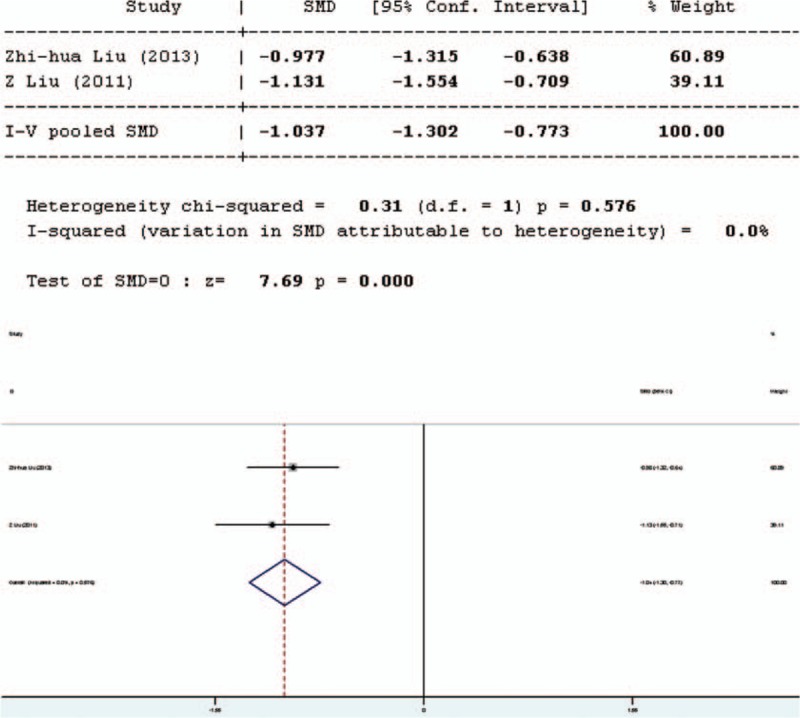
Forest plot for effect of pro-/synbiotics on lactulose/mannitol test in patients with colorectal resection (in English). CI = confidence interval, ID = identification, SMD = standardized mean difference.

**FIGURE 3:**
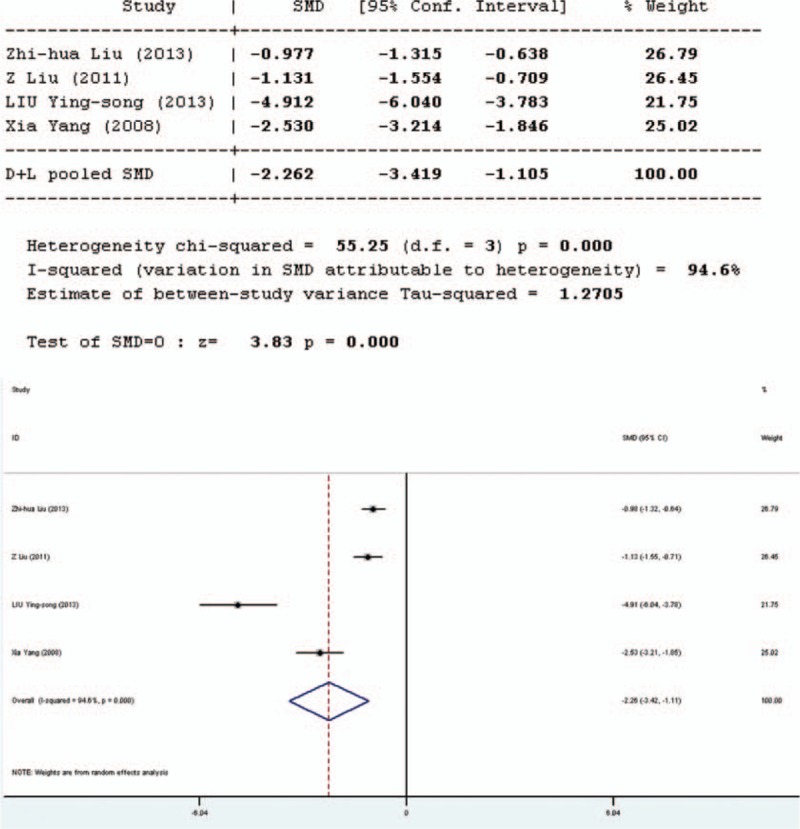
Forest plot for effect of pro-/synbiotics on lactulose/mannitol test in patients with colorectal resection (all articles). CI = confidence interval, ID = identification, SMD = standardized mean difference.

Intestinal permeability was assessed by using the lactulose/mannitol (L/M) test. Four studies involved in the meta-analysis provided applicable L/M test data, 2 in English and 2 in Chinese. Two separate analyses have been performed, one including only English articles (Figure 2), and the other including all articles. The forest plot of the results of the articles in English (Figure 3) showed that the 2 results had good homogeneity for the L/M test (*Q* = 0.31, d*f* = 1, *P* = 0.576, *I*^2^ = 0.0%) and fixed-effects model was used. The results of the fixed-effects model demonstrated significant difference in L/M test results between the pro-/synbiotics group and the control group (standardized mean difference = 7.69, *P* = 0.000). The forest plot of the results of all articles (Figure 3) showed that the result for heterogeneity was significant for the L/M test (*Q* = 55.25, d*f* = 3, *P* = 0.0000, *I*^2^ = 94.6%). The 4 studies were heterogeneous and the random-effects model was used. The results of the random-effects model demonstrated significant difference in L/M test results between the pro-/synbiotics group and the control group (standardized mean difference = 3.83, *P* = 0.000). The permeability of the intestinal mucosa in the treatment group was better than that in the control group.

#### Occludin

**FIGURE 4:**
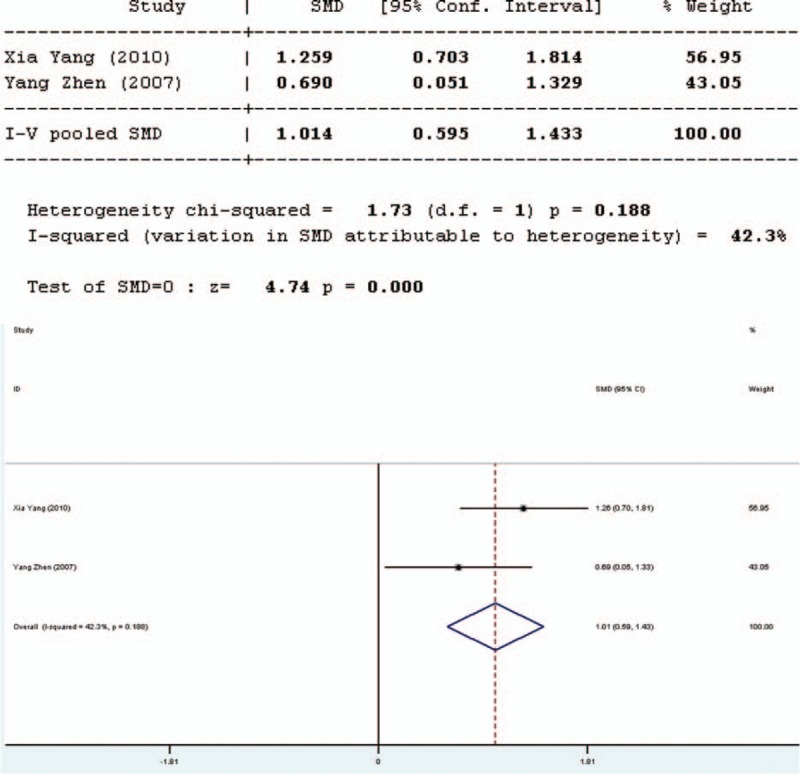
Forest plot for effect of pro-/synbiotics on occludin in patients with colorectal resection (all articles). CI = confidence interval, ID = identification, SMD = standardized mean difference.

Occludin was used to assess the intestinal mechanical barrier function. Three studies involved in the meta-analysis provided applicable data concerning occludin. However, the data of one English study were not complete; the remaining 2 studies in Chinese involved in the meta-analysis provided applicable data on occludin. The forest plot of the results of all articles (Figure 4) showed that the 2 reports had good homogeneity (*Q* = 1.73, d*r* = 1, *P* = 0.188, *I*^2^ = 42.3%). It was shown that there was significant difference in occludin between the pro-/synbiotics group and the control group (standardized mean difference = 4.74, *P* = 0.000). The study in English showed that the occludin level in the experimental group was higher than that in the control group (*P* < 0.05). The occludin level in the experimental group was significantly higher than that in the control group.

#### *Bifidobacterium* to *Escherichia* Ratio

**FIGURE 5:**
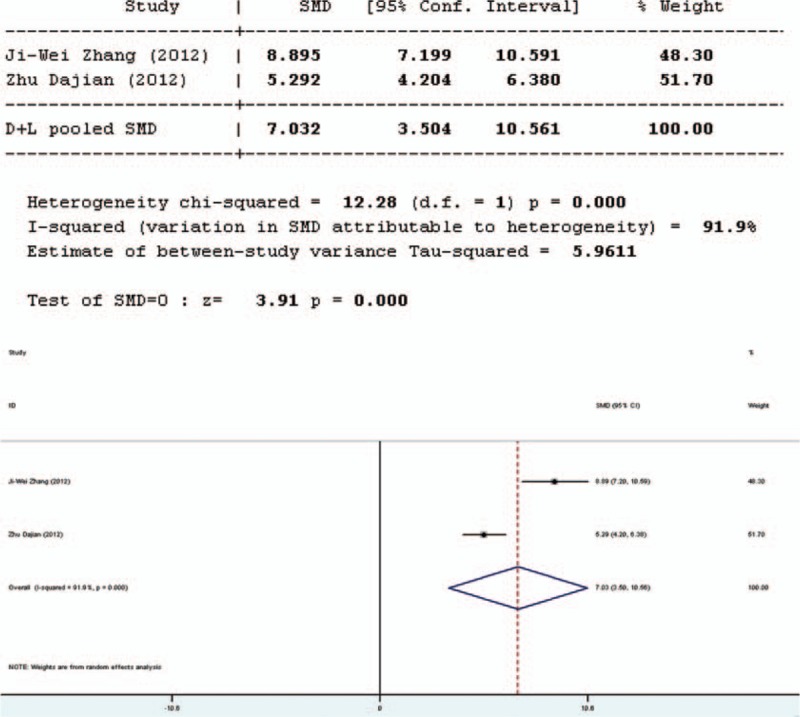
Forest plot for effect of pro-/synbiotics on *Bifidobacterium* to *Escherichia* ratio in patients with colorectal resection (all articles).

The ratio of *Bifidobacterium* to *Escherichia* (B/E) was used to assess microbial colonization resistance. Three studies involved in the meta-analysis provided applicable data on B/E. However, 2 of them were duplicate publications. Only the more recent data were selected. Therefore, only 2 studies were included in the evaluation of B/E, 1 in English and 1 in Chinese. The forest plot of the results of all articles (Figure 5) showed that the test result for heterogeneity was significant for B/E (*Q* = 12.28, d*f* = 1, *P* = 0.000, *I*^2^ = 91.9%). Heterogeneity was found between the 2 studies and the random-effects model was used. The results of the random-effects model indicated that there was significant difference in B/E between the pro-/synbiotics group and the control group (standardized mean difference = 3.91, *P* = 0.000). The study in English showed that the B/E of the experimental group was higher than the control group (*P* < 0.001). Therefore, the B/E of the experimental group was higher than the control group.

### Bacterial Translocation

**FIGURE 6:**
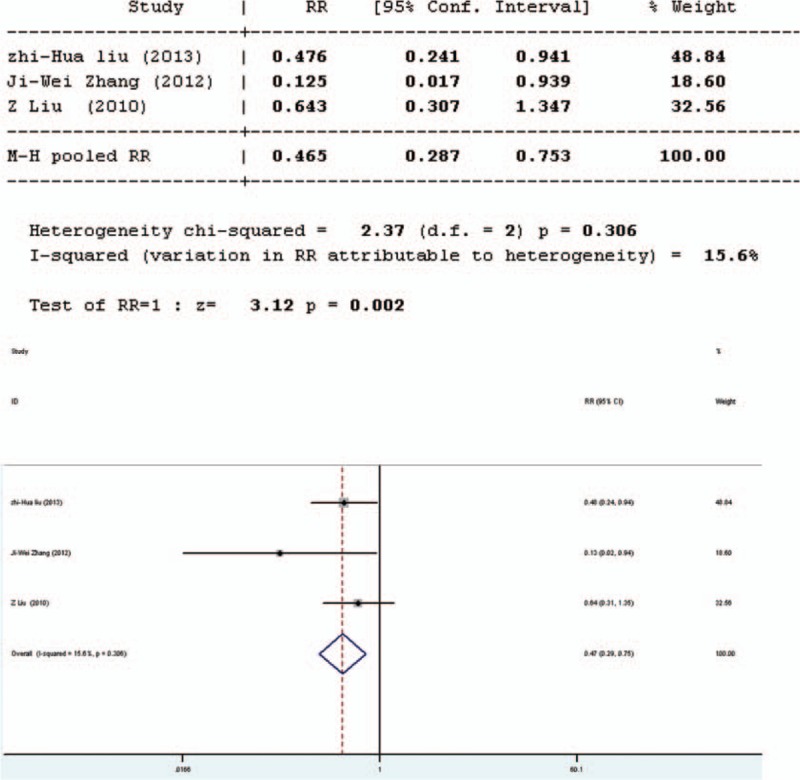
Forest plot for effect of pro-/synbiotics on bacterial translocation in patients with colorectal resection (all articles). CI = confidence interval, ID = identification, SMD = standardized mean difference.

Bacterial translocation can also be used to assess microbial colonization resistance. Three studies involved in the meta-analysis provided applicable data on bacterial translocation which were all in English. The forest plot of the results (Figure 6) showed that the 3 studies had good homogeneity (*Q* = 2.37, *P* = 0.306, *I*^2^ = 15.6%). It was suggested that there was significant difference in bacterial translocation between the pro-/synbiotics group and the control group (standardized mean difference = 3.12, *P* = 0.002). The bacterial translocation rate in the experimental group was significant lower than that in the control group (*P* = 0.002).

### Secretory Immunoglobulin A

**FIGURE 7:**
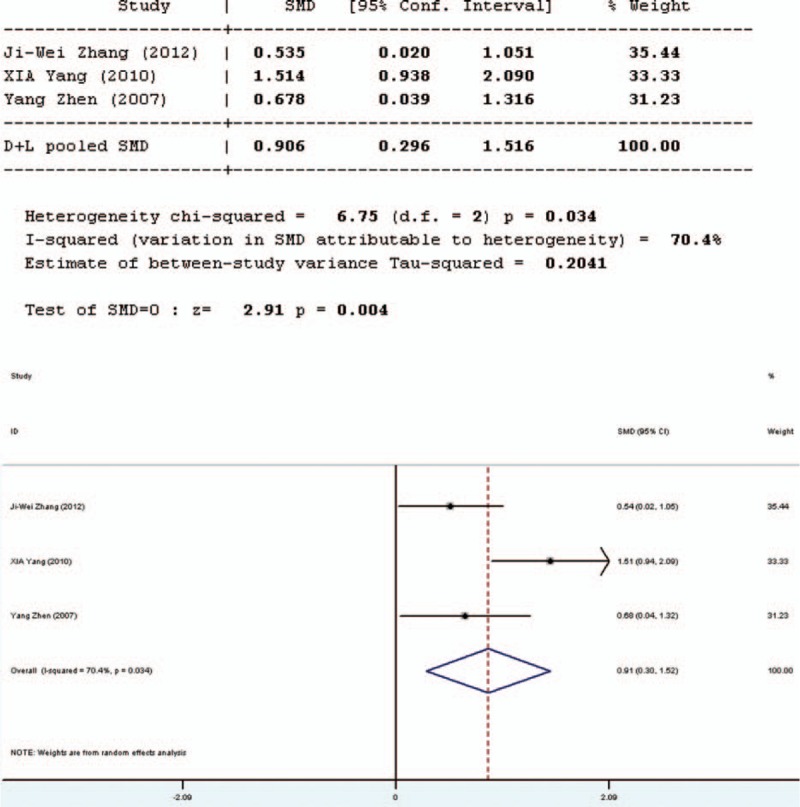
Forest plot for effect of pro-/synbiotics on secretory immunoglobulin A in patients with colorectal resection (all articles). CI = confidence interval, ID = identification, SMD = standardized mean difference.

Four studies involved in the meta-analysis provided applicable data on secretory immunoglobulin A (SIgA). However, 2 of them were duplicate publications. Only the more recent data were selected. Therefore, only 3 studies were included in the evaluation of SIgA, 1 in English and 2 in Chinese. The forest plot of the results of all articles (Figure 7) showed that the test results for heterogeneity were significant for SIgA (*Q* = 6.75, d*f* = 2, *P* = 0.013, *I*^2^ = 70.4%). The 3 studies were heterogeneous and the random-effects model was used. It was revealed in the results of the random-effects model that there was significant difference in SIgA between the pro-/synbiotics group and the control group (standardized mean difference = 2.91, *P* = 0.004). The study in English showed that the amount of SIgA in the experimental group was higher than that in the control group. Therefore, the amount of SIgA in the experimental group was higher than that in the control group.

### Interleukin-6

**FIGURE 8:**
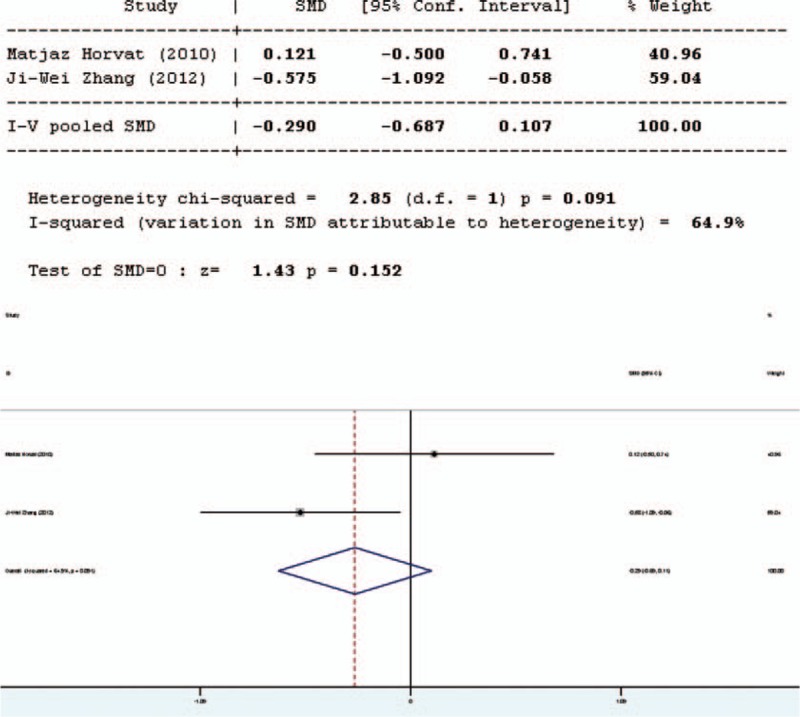
Forest plot for effect of pro-/synbiotics on interleukin-6 in patients with colorectal resection (all articles). CI = confidence interval, ID = identification, SMD = standardized mean difference.

**FIGURE 9:**
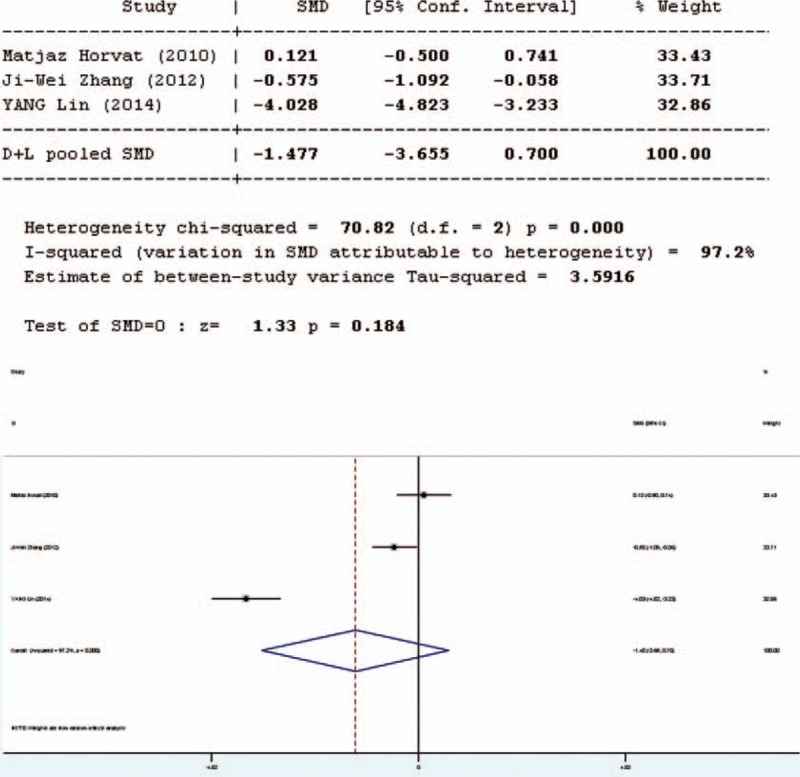
Forest plot for effect of pro-/synbiotics on interleukin-6 in patients with colorectal resection (all articles). CI = confidence interval, ID = identification, SMD = standardized mean difference.

Five studies involved in the meta-analysis provided applicable data on interleukin-6 (IL-6). However, 2 of them were duplicate publications. Only the more recent data were selected. Moreover, the data of one study were not complete. Thus, only 3 studies were included in the evaluation of IL-6, 2 in English and 1 in Chinese. The forest plot of the results of the articles in English (Figure 8 Forest plot for effect of pro-/synbiotics on IL-6 in patients with colorectal resection (In English)] showed that the two results had good homogeneity for the IL-6 test (*Q* = 2.85, d*f* = 1, *P* = 0.091, *I*^2^ = 64.9%) and fixed-effects model was used. The results showed that there was no significant difference in the amount of IL-6 between the pro-/synbiotics group and the control group (standardized mean difference = 1.43, *P* = 0.152). The forest plot of the results of all articles (Figure 9) showed significant heterogeneity were for IL-6 (*Q* = 70.82, d*f* = 2,*P* = 0.000, *I*^2^ = 97.2%). The 3 studies were heterogeneous and the random-effects model was used. The results showed that there was no significant difference in the amount of IL-6 between the pro-/synbiotics group and the control group (standardized mean difference = 1.33, *P* = 0.184). Therefore, the amount of IL-6 between the 2 groups had no significant differences.

### C-reactive Protein

**FIGURE 10:**
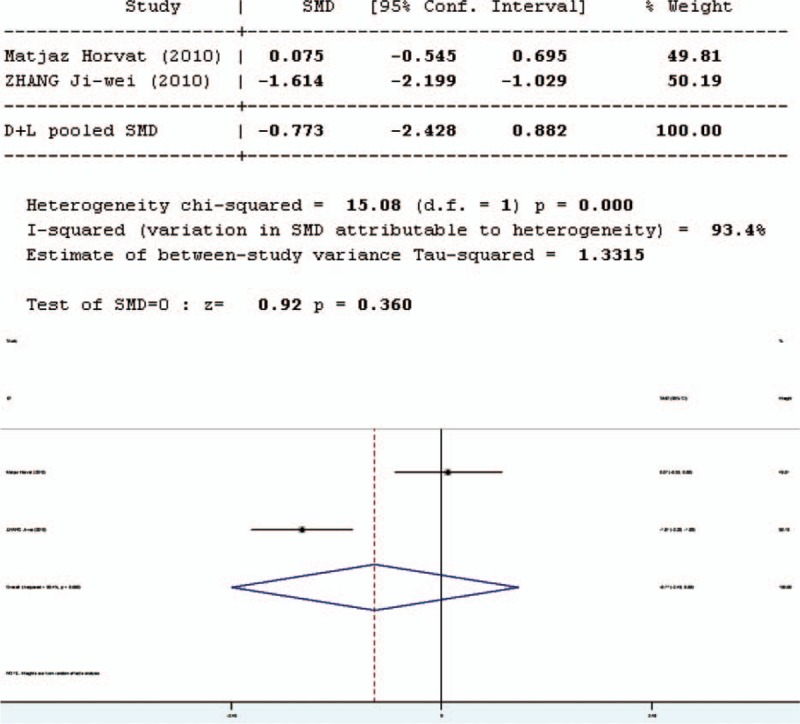
Forest plot for effect of pro-/synbiotics on CRP in patients with colorectal resection (in English). CI = confidence interval, ID = identification, SMD = standardized mean difference.

Intestinal stress status was assessed by the C-reactive protein (CRP) test. Ten studies involved in the meta-analysis provided data on CRP. However, the data of 1 study were not complete. Therefore, 9 studies involved in the meta-analysis provided applicable data on CRP, 2 in English and 7 in Chinese. The forest plot (Figure 10) of the results of the articles in English showed that the test result for heterogeneity was significant in CRP (*Q* = 15.08, d*f* = 1, *P* = 0.0000, *I*^2^ = 93.4%). There was heterogeneity in the 2 studies and the random-effects model was used. The results of the random- effects model showed that there was no significant difference in CRP between the pro-/synbiotics group and the control group (standardized mean difference = 0.92, *P* = 0.36).

**FIGURE 11:**
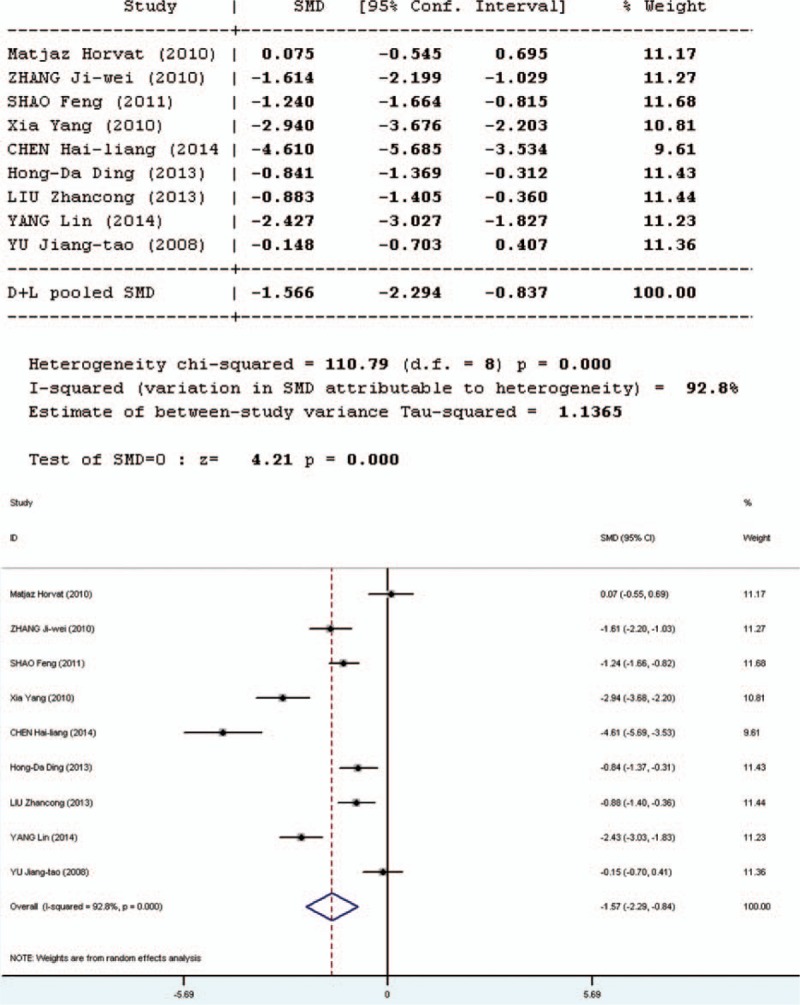
Forest plot for effect of pro-/synbiotics on C-reactive protein in patients with colorectal resection (all articles). CI = confidence interval, ID = identification, SMD = standardized mean difference.

The forest plot (Figure 11) of the results of all articles showed that the test result for heterogeneity was significant in CRP (*Q* = 110.79, d*f* = 8, *P* = 0.0000, *I*^2^ = 92.8%). There was heterogeneity in the 9 studies and the random-effects model was used. The results of the random-effects model showed that there was significant difference in CRP between the pro-/synbiotics group and the control group (standardized mean difference = 4.21, *P* = 0.000). The level of CRP in the experimental group was lower than that in the control group. Therefore, the effect of probiotics on CRP is not certain.

### Sensitivity Analysis and Publication Bias

After excluding low-quality trials (ie, studies with a Jadad score <3) and the study with the minimum sample size, the results of the remaining 5 studies were analyzed. These results were similar to the previous results, indicating that the results of meta-analysis were relatively credible. Publication bias could not be estimated with Begg funnel plot, owing to the restricted number of trials included in this meta-analysis.

## DISCUSSION

The gut is the body's biggest bacterial and toxin reservoir. With the complete barrier function under normal conditions, the intestinal tract can protect the body from toxins, bacteria, and other damage. The complete intestinal mucosal barrier includes physical, immunological, biological, and chemical barriers.^28^ Owing to tumors, the intestinal tract may suffer different degrees of intestinal microecology disorder in patients with colorectal cancer. Banning diet and drinks postoperatively, using antibiotics, parenteral nutrition, and antacids can destroy the intestinal microbial ecological balance, intestinal mucosal barrier permeability, and intestinal mucosal immune function. Therefore, these patients are prone to develop functional diarrhea, infectious diarrhea, bacterial flora imbalance, bacterial flora shift, endotoxin, intestinal infection, etc.^29^ Probiotics can promote the intestinal microflora balance and have a beneficial effect on the host. It is demonstrated in one study^30^ that probiotics can promote the expression of tight junction proteins in intestinal epithelial cells, mucin, and intestinal epithelium, and enhance the function of the intestinal epithelial cell barrier. Probiotics can promote mucus secretion of the epithelial cells to form a protective layer between the mucosa and microbes, thus preventing bacteria and toxin translocation and inhibiting pathogen growth, as well as adhesion and invasion of the pathogenic bacteria to the intestinal epithelial cells.^31^ Therefore, the use of probiotics for the rehabilitation of patients with colorectal cancer after surgery is important.

First, the L/M and occludin tests are used to evaluate the physical barrier. The physical barrier is composed of a mucous layer, a layer of epithelium cells, and the directional peristalsis of the intestinal mucosa.^32^ Lactulose is a disaccharide with a relative molecular weight of 342 Da (diameter, 0.95 nm), which is nontoxic and metabolized in vivo. Lactulose can be accurately measured in urine after intravenous injection and is absorbed by the cells. Mannitol is a monosaccharide with a relative molecular weight of 182 Da (diameter 0.67 nm). It is water-soluble and can be absorbed by micropores in the membrane of the epithelium. When the intestinal mucosal barrier function is impaired, the intestinal mucosa atrophies and the tight connection between the cells and the cell gap increases. The absorption of lactulose increases, but the absorption of mannitol by the cell pathway is not changed obviously. So the ratio of L/M increases. At the mean time, the effects on lactulose and mannitol of gastrointestinal motility and kidney emptying are almost equal. Thus, the ratio of lactulose to mannitol can be used to evaluate intestinal mucosa permeability.^33^ It was shown in this study that the L/M ratios of the intestinal mucosa in the experimental group were lower than the control group, which means that the intestinal permeability of the experimental group was lower than that of the control group. Occludin is a cellular membrane protein. It usually contains a transmembranous structure and is one of the main structural proteins that constitute the mechanical barrier. Therefore, the level of occludin can reflect the function of the intestinal mechanical barrier.^28^ The results of this study showed that the level of occludin in the experimental group was higher than that in the control group. Probiotics can improve the expression of occludin in the intestinal epithelium. Therefore, probiotics can protect the intestinal mucosal permeability by maintaining normal physical barrier function.

Second, the intestinal biological barrier is composed of the colonization resistance by normal synbiotic bacteria in the intestine and the accumulation of bacteria.^34^ The intestinal biological barrier can protect against the adhesion and propagation of Gram-negative aerobic bacteria, and reduce bacterial translocation.^31^ However, some bacteria, such as *Escherichia coli*, can promote the formation of carcinogens, such as 2-methylhydrazine and nitrite, by secreting β-glucose and azo reductase, thereby inducing intestinal tumors.^35^ Intestinal probiotics, such as *Lactobacillus*, play an important role in antitumor activity by stimulating the host's immune activity. Thus, the intestinal flora can be either a tumor promoter or tumor suppressor, depending on the type of bacteria. And the ratio of *Bifidobacterium* to *Escherichia* is usually used to evaluate the intestinal flora. An inversion in the ratio of *Bifidobacterium* to *Escherichia* indicates a flora imbalance in the intestinal tract. The growth and propagation of probiotics would be disturbed, and the intestinal resistance to colonization of pathogenic bacteria is weakened. The numbers of anaerobic bacteria, represented by *Bifidobacterium*, in the perioperative period of colorectal cancer significantly decrease, and the numbers of aerobic bacteria, represented by *Escherichia coli*, increase significantly. In this study, the ratio of B/E was significantly different between the treatment group and the control group, with the changes in B/E more significant in the control group than the treatment group. Therefore, it can be deduced that probiotics can inhibit the flora imbalance in the perioperative period of colorectal cancer. In addition, the bacterial translocation rate was used to evaluate the biological barrier function. Bacterial translocation refers to the transfer of intestinal bacteria and their products from the intestinal lumen to the mesentery or other intestinal organs. Under normal circumstances, intestinal bacterial translocation is low, owing to the strong intestinal mucosal barrier. The epithelium of the intestinal mucosa is closely linked, and has good capacity for surface bacterial clearance. The intestinal mucosa permeability and bacterial translocation rate increase when the intestinal lesions are stressed. Therefore, bacterial translocation can be used to evaluate the intestinal mucosal barrier permeability and the intestinal mucosal biological barrier. The results showed that the bacterial translocation rate of the experimental group was lower than that of the control group, suggesting that probiotics can significantly reduce the bacterial translocation rate of the intestine, thus protecting the intestinal bacterial barrier. Therefore, probiotics can increase the intestine's resistance to pathogenic bacteria colonization by reducing both intestinal bacteria dysfunction and the bacterial translocation rate, thereby protecting the intestinal biological barrier, to inhibit tumors and reduce postoperative intestinal complications.

The immune barrier is mainly composed of the SIgA immune system and T cell immune system.^30^ Therefore, SIgA, CRP, and IL-6 can be used to evaluate the immune barrier. As the main immunoglobulin in the intestine, SIgA has long been considered the first-line immune defense, offering resistance to invading pathogens in the mucosa. It can prevent bacteria from adhering to the surface of epithelial cells, has an antiviral effect, neutralizing toxins and other biological active antigens, and has a wide range of immune protection. Thus, the abnormal secretion of SIgA is bound to weaken the immune function in the intestine.^36,37^ The results of this study showed that the amount of SIgA secreted by the experimental group was higher than that of the control group. It suggests that probiotics can increase the amount of intestinal SIgA secretion and help colon cancer patients to maintain the intestinal immune barrier. Moreover, CRP was also used to evaluate the intestinal mucosal immune barrier. CRP is a kind of acute-phase reactive protein and can be raised by proinflammatory cytokine IL-6 and tumor necrosis factor (TNF).^38^ The concentration of CRP was <l0 μg/mL in normal conditions. When tissue injury and acute infection occur, the serum concentration of CRP elevates sharply in hours, reaches a peak in 24 to 48 hours, and returns to normal when symptoms begin to disappear. CRP could be detected earlier than other acute-phase reactants, and is not affected by radiotherapy, chemotherapy, or corticosteroids.^39^ As a result, it is a good indicator of inflammation. The mechanism that causes the increase in serum concentrations of CRP in cancer patients is not clear. It may be resulted from an increased level of serum cytokines IL-6 and TNF in cancer patients, which would stimulate the liver to synthesize CRP.^40,41^ Therefore, CRP can be used as an index of the intestinal immune function for the prognosis of patients with colorectal cancer, and to observe the therapeutic effects of probiotics on the immune function. In this study, there was no difference of CRP between the 2 groups in the results of the English articles. But there was a significant difference between the 2 groups in the results of all articles, indicating that the role of probiotics in regulating CRP was not certain. The study also used cytokine IL-6 to evaluate the intestinal immune function. Cytokines are the major regulators of mucosal immunity, and play the same important role in intestinal immune defense. In the intestinal lesion or stressed state, T cell activation releases TNF-α, thus inducing the generation of IL-1 and IL-6, protecting the intestinal mucosal immune barrier, increasing intestinal mucosa permeability and promoting bacterial translocation.^28^ It is shown in this study that there was no significant difference in IL-6 secretion in the experimental group compared with the control group. It is manifest that probiotics cannot reduce the secretion of IL-6 and the damage of intestinal immune barrier through intestinal lymphoid tissue and the mucous membrane. The function of probiotics in the protection of intestinal normal immune barrier is not certain, and that further studies are needed.

There are some limitations to this meta-analysis. First, the retrieved studies were significantly heterogeneous, and the type and concentration of the probiotics, duration of therapy (including optimal dose, time, and type), and the therapeutic route (ie, combination of perioperative, postoperative, or preoperative use versus no use) varied considerably among the included studies. As there was no enough number of eligible studies, analyses of regression and publication bias could not be conducted. In addition, some of the included studies had small sample sizes, which might influence the reliability and validity of the conclusions. Finally, only including studies published in the language of Chinese and English was another possible limitation.

In conclusion, it is indicated in this research that the use of probiotics in patients undergoing colorectal resection is a promising protective measure that might effectively protect the physical and biological barrier of the gastrointestinal mucosa. However, there was no obvious effect in the reduction of IL-6 secretion. Well-designed RCTs are needed to further explore potential mechanisms of action, to optimize the use of probiotics, and assess the efficacy and safety of probiotics or synbiotics in protecting the gastrointestinal mucosa immune barrier.
